# Thermal referral: evidence for a thermoceptive uniformity illusion without touch

**DOI:** 10.1038/srep35286

**Published:** 2016-10-24

**Authors:** Antonio Cataldo, Elisa Raffaella Ferrè, Giuseppe di Pellegrino, Patrick Haggard

**Affiliations:** 1 Institute of Cognitive Neuroscience, University College London, UK; 2 Centre for studies and researches in Cognitive Neuroscience, University of Bologna, Italy; 3 Department of Psychology, Royal Holloway University of London, UK

## Abstract

When warm thermal stimulators are placed on the ring and index fingers of one hand, and a neutral-temperature stimulator on the middle finger, all three fingers feel warm. This illusion is known as thermal referral (TR). On one interpretation, the heterogenous thermal signals are overridden by homogenous tactile signals. This cross-modal thermo-tactile interaction could reflect a process of object recognition, based on the prior that many objects are thermally homogenous. Interestingly, the illusion was reported to disappear when the middle digit was lifted off the thermal stimulator, suggesting that tactile stimulation is necessary. However, no study has investigated whether purely thermal stimulation might induce TR, without any tactile object to which temperature can be attributed. We used radiant thermal stimulation to deliver purely thermal stimuli, which either were or were not accompanied by simultaneous touch. We found identical TR effects in both the original thermo-tactile condition, and in a purely thermoceptive condition where no tactile object was present. Control experiments ruled out explanations based on poor spatial discrimination of warm signals. Our purely thermoceptive results suggest that TR could reflect low-level organization of the thermoceptive pathway, rather than a cognitive intermodal modulation based on tactile object perception.

The somatosensory system comprises several submodalities, based on distinct peripheral receptor types. Submodality specificity is preserved in peripheral afferent fibres. However, complex central interactions between submodalities also occur, first within the spinal cord and then in the brain^1^. Here we focus on interactions between touch and temperature. This interaction remains controversial because these submodalities have distinct cortical targets (primary somatosensory cortex and insula, respectively^2^), yet the perception of touch and temperature are strongly interdependent^3^,^4^,^5^. Indeed, somatic experiences often have a unitary thermo-tactile quality suggesting an obligatory cross-modal interaction: while holding a hot cup of tea, it is impossible to dissociate perceptually the touch of the cup from the warm sensation.

The Thermal Referral (TR) phenomenon is a striking demonstration of this thermo-tactile interaction^4^,^5^,^6^. When innocuous warm thermal stimulators were applied to the ring and index fingertips of one hand, and a neutral-temperature stimulator to the middle finger, all three fingers felt warm. That is, the thermal sensation at the outer fingers was referred to the middle finger. Similar TR phenomena were found for cold stimuli. As a consequence, a pattern of physical stimulation that was tactually uniform, but thermally non-uniform, was illusorily perceived as thermally uniform^5^. At the same time, the perceived overall intensity was reduced, relative to a condition where all three fingers were actually stimulated^5^. According to a classic account of TR, the tactile system signals homogeneity, because the mechanical contact of finger and stimulator is common to all three fingers, while the peripheral thermal system signals heterogeneity, with different temperatures at each finger. In integrating these signals to provide a multisensory percept of the thermo-tactile object, tactile information is given a higher weighting than thermal information, so thermal signals specific to each finger are lost to perception^4^,^5^,^6^. Ho and colleagues^5^ recently proposed an account based on serial processing, rather than an integration of parallel temperature and touch signals. At a first stage, spatial summation^7^,^8^ tends to homogenize thermal percepts across multiple stimulated areas, producing an overall intensity percept proportional to the stimulated area. At a second stage, this intensity is then referred or attributed, on the basis of touch, to individual body parts. On this view, TR is a cross-modal phenomenon, in which tactile input on the middle finger drives the illusory perception of warmth.

Strong evidence for the role of touch in TR comes from reports that illusory thermal sensations disappeared when the middle digit was lifted off the thermal stimulator^4^. The actual state of the middle finger was thermally neutral with and without tactile contact, but the perceived temperature was warm during tactile contact, and thermally neutral without it. This result also seems to rule out explanations based merely on strong spatial summation within the thermoceptive system, since summation should ensure a continued perception of warmth, perhaps with some modest decrease depending on the strength of summation. These results suggested that tactile information is essential for TR.

However, to our knowledge, no study has tested whether purely thermal stimulation, without any tactile stimulation at all, can also induce TR. An affirmative result would cast doubt on the standard interpretation of TR as a cross-modal perceptual illusion driven by tactual object perception, and point instead to spatial interactions within the thermoceptive system. We accordingly developed a novel radiant thermal apparatus that allowed us to deliver either thermo-tactile or purely thermal stimuli. We replicated classical TR results regarding uniformity (experiment 1) and intensity of thermal perception (experiment 2) in a thermo-tactile condition. Crucially, we observed a purely thermoceptive version of TR in the absence of any tactile stimulation, which reproduced the features previously described for classical thermo-tactile TR. We also demonstrated that the thermoceptive version of TR cannot merely ascribed to poor thermal resolution (experiment 3).

## Methods

### Participants

Thirteen healthy right-handed participants (10 female, mean age ± SD: 23.8 ± 3.1 years) took part in experiment 1. One participant was excluded because of inability to follow instructions (see below). A group of twelve new participants volunteered in experiment 2 (10 female, mean age ± SD: 24.6 ± 3.9 years), and a further twelve new participants (9 female, mean age ± SD: 25.4 ± 5 years) volunteered in experiment 3. The sample size for each experiment (n = 12) was decided a priori on the basis of previous similar studies. The experimental protocol was approved by the research ethics committee of University College London. The study adhered to the ethical standards of the Declaration of Helsinki. All participants provided their written informed consent before the beginning of each experiment.

### Radiant Thermal Stimulation

**Figure 1:**
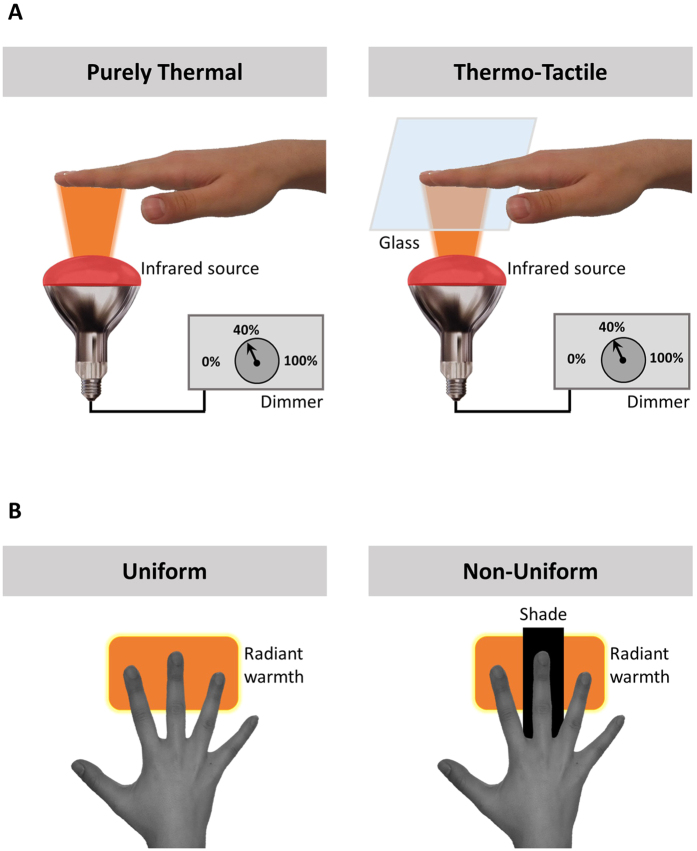
Experimental set up and conditions. (**A**) Thermal radiant stimuli were delivered by a 125 mm diameter, 250 watt infrared light bulb using three different stimulation intensities by connecting the bulb to one of three dimmer switches preset at 0% (no stimulation), 40% (low intensity), and 100% (high intensity). The participant’s right hand rested above the infrared source. Intermediate and distal phalanges of the index, middle and ring fingers were exposed to the thermal stimulation. In the thermo-tactile condition, the fingers rested on a sheet of glass. (**B**) To generate the non-uniform condition, an aluminium shade was placed between lamp and middle finger, to cast a heat shadow over the middle finger.

Figure 1 illustrates the experimental set-up used in the three experiments. Thermal radiant stimuli were delivered by a 125 mm diameter, 250 watt infrared light bulb. Three different stimulation intensities were delivered, by connecting the bulb to one of three dimmer. The switches were set at 0% (no stimulation), 40% (low intensity), and 100% (high intensity) of their range, respectively, and were not further adjusted during the experiment. These non-zero intensities were selected to produce transient increases in skin temperature that were higher than the thermal detection threshold of the hand (i.e., >1 °C)^9^, lower than pain threshold^10^,^11^, but readily discriminable between all three levels.

**Figure 2:**
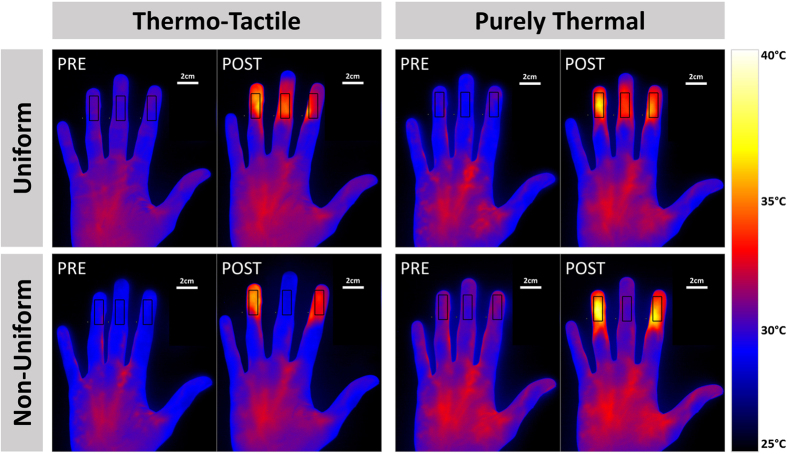
Thermographic images. Thermal infrared imaging data recorded in a participant. A thermographic camera was used to film the entire experimental procedure. Two single frames were extracted, depicting the thermal profile of the hand immediately before and after warm radiant stimulation. A region of interest corresponding to the area of the skin exposed to the stimulation was marked on each finger. The change in temperature for each experimental condition was computed as the difference between post- and pre-stimulation mean temperature within each region of interest. Uniform pattern of stimulation (top row) induced an overall increase in temperature in all finger, with no differences between the middle finger and the outer fingers (middle: 4 °C; outer fingers: 3.8 °C, tactile conditions averaged). Conversely, the non-uniform warm-neutral-warm patter (bottom row) triggered a selective increase in temperature in the outer fingers, while the temperature of the shaded middle finger did not change (middle: 0.1 °C; outer fingers: 4.5 °C, tactile conditions averaged).

The participant’s right hand was placed 11 cm above the infrared source, pronated on a moulded support. This support left the intermediate and distal phalanges of the index, middle and ring fingers exposed, while shielding the rest of the hand. In particular, the support blocked the radiant heat from reaching the thumb and the little finger. Two layers of 2 mm of thickness crystal glass were placed between the hand and the source. This allowed thermal radiant stimulation of the fingers, while isolating the fingers from potential air convection surrounding the infrared source. The upper glass was replaced after each trial to prevent it from overheating, and becoming an additional source. In the thermo-tactile condition, the upper glass sheet was raised until it contacted the glabrous skin of the index, middle and ring fingers, creating a 3 mm gap between the glasses, and providing further thermal isolation. To generate the neutral middle finger temperatures associated with TR, we placed a 4 × 12 cm aluminium shade between the two layers of glass, thus casting a heat shadow over the middle finger. Additional vertical aluminium spacers between index and middle fingers, and between middle and ring fingers prevented any radiant heat stimulation of the middle finger from above the upper glass. Accurate stimulus delivery was validated by measuring actual skin temperature in each condition of each experiment with a spot infrared thermometer (Precision Gold, N85FR Maplin, UK) (see below). We additionally used an infrared thermal camera (FLIR Silver SC5000 MWIR, FLIR systems, Oregon, USA) to quantify the effects of our thermal stimulation in one participant (who did not take part in any other experiment). The analyses focused on the spatial specificity of thermal stimulation, and its profile over time. These images confirmed that our apparatus could selectively warm some fingers, without inducing any substantial temperature changes of other adjacent fingers that were shielded from the radiant heat source (see Fig. 2 for details).

### Experiment 1

#### Individual finger temperature perception task

Each participant completed two different tasks in a fixed order. The first task was a thermal perception task, which served both as a validation of the stimulation method and as perceptual calibration of thermoception on each finger. The second task aimed to replicate Ho and colleagues^5^ uniformity judgement method for investigating TR (see later).

One of three thermal radiant stimulation intensities (no stimulation, low intensity, high intensity) was delivered to the index, middle or ring finger of the right hand, in both a thermo-tactile and a purely thermal condition. At the beginning of each trial, the participant placed his right hand for 20 seconds in a 31 °C thermostatic water bath to set skin temperature at a constant baseline level. Skin temperature was measured by an infrared thermometer, and found to conform to the intended baseline (range: 28 °C–32 °C; mean baseline temperature ± SD: 29.9 °C ± 1 °C). Next, the experimenter dried the hand quickly, and placed it on the support. Radiant thermal stimulation was delivered to the target finger for 15 seconds based on pilot tests. This duration reliably increased skin temperatures, and also produced a clear detectable warmth sensation. Importantly, the stimulation was always below pain threshold. A sound signaled the end of the stimulation, after which the participant made a verbal response and the experimenter measured again skin temperature (post-stimulation). Participants were asked to rate the intensity of the thermal stimulation from 0 (no stimulation) to 10 (very hot). One stimulation at maximum intensity was given at the beginning of the experiment, and participants were instructed that experimental stimulations would always be below this level. This gave a functional anchor for the judgement scale. Each combination of three intensities, three fingers and two tactile conditions was repeated twice, giving 36 stimulations in total. Finger stimulated and intensity of stimulation were randomized within participant, while tactile condition order was counterbalanced between participants. Participants were blindfolded for the entire duration of the task.

#### Thermal uniformity perception

Uniformity judgement procedure was based on previous reports^5^ and on the stimulation methods described above. Radiant thermal stimuli were delivered on the right index, middle and ring fingers, and participants judged the uniformity of the stimulation across all three fingers, by verbally responding “uniform”/“non-uniform”. In the non-uniform condition, a shade with two vertical spacers was interposed between the infrared lamp and the middle finger, while leaving the outer fingers exposed to the infrared light. In the uniform condition, a similar object composed by the two vertical spacers only, and no shade, was placed among the fingers, in order to match any auditory cue related to the application of the shade in the non-uniform condition. The low (40% of maximum) and high (100% of maximum) stimulation intensity levels of the previous task were used. Skin temperature was also recorded pre- and post-stimulation using an infrared thermometer.

Participants were asked to report whether the stimulation was uniform across all three fingers or not. Thermo-tactile stimulation conditions, and purely thermal conditions were both tested. Intensity (low/high) and spatial pattern (uniform/non-uniform) of stimulation were randomized within participant, while the order of tactile condition (thermo-tactile/purely thermal) was blocked counterbalanced between participants. Each stimulus was repeated five times, giving a total of 40 stimulations. For the entire duration of the task participants were blindfolded.

### Experiment 2

#### Thermal intensity perception

In this experiment we measured the perceived intensity of the sensation resulting from TR. Previous studies reported a decrease in the overall perceived intensity in the thermo-tactile non-uniform patterns compared to spatially uniform patterns^5^. This decrease was used as evidence that total thermal stimulation was redistributed across relevant tactile inputs. Here we investigated whether a similar reduction in the perceived overall intensity is present in the purely thermal TR.

The intensity perception task procedure was based on previous reports^5^ and the stimulation methods described above. We quantified intensity perception using temperature matching^5^,^12^. In particular, we chose matching temperature as a dependent variable, because it gives continuous, quantitative data, is commonly reported in somatosensory sensations^13^, has been reliably used before in matching tasks^14^, and reflects the same continuous, underlying mechanism as thermal judgement. Participants were asked to place their right hand over a support, which allowed radiant thermal stimulation of the index, middle and ring fingers. Stimulation and temperature measurement were as in experiment 1. A 13 mm diameter thermode (Physitemp Instruments Inc, NTE-2A, New Jersey, USA) was mounted on a stand touching the participant’s forehead. A chinrest ensured a constant contact and pressure between the thermode and the skin. The temperature of this probe was initially set at 30 °C. After 10 seconds from the beginning of the thermal radiant stimulation, the temperature of the forehead thermode was increased at +0.5 °C/s. Participants were instructed to report by a keypress when the forehead temperature matched the perceived temperature of the three stimulated fingers of the right hand. The radiant stimulation duration was set so that this was expected to occur after approximately 15 seconds, matching the stimulation durations in experiment 1.

Intensity (low/high) and spatial pattern (uniform/non-uniform) of stimulation were randomized within participant, while the order of tactile condition (thermo-tactile/purely thermal) was counterbalanced between participants. Each stimulus was repeated five times, giving a total of 40 stimulations. For the entire duration of the task participants were blindfolded and kept their forehead in contact with the thermode.

### Experiment 3

#### Thermal spatial localization

Localization of thermal stimuli on the skin is reported to be poor^15^,^16^. Therefore, referred sensations in our experiments might potentially be driven by mislocalisation of thermal stimuli across the fingers, rather than by TR-like mechanisms. We therefore delivered radiant heat stimuli to a *single* finger, and investigated participants’ ability to identify the stimulated finger.

The procedure was based on methods described above. Low and high intensity purely thermal stimuli were randomly delivered to the index, middle or ring finger of participants’ right hand, without any tactile stimulation. Only one finger was stimulated during each trial. Pre- and post- skin temperature for the stimulated finger was recorded. After 15 seconds of thermal stimulation, participants verbally reported which finger was stimulated. Intensity (low/high) and position (index/middle/ring) of stimulation were randomized within participants. Each stimulus was repeated five times, giving a total of 30 trials. For the entire duration of the task participants were blindfolded.

## Results

### Experiment 1

#### Individual finger temperature perception task

We focused on whether the thermal radiant stimulation delivered was effective and reliable. First, we checked whether the radiant thermal stimuli produced a measurable increase in skin temperature. We computed the difference between post-stimulation and pre-stimulation skin temperature, and directly compared the temperature gain for no stimulation vs low intensity, and then for low vs high intensity stimulation. Clear differences in skin temperature were found for each finger in both thermo-tactile (no stimulation vs low intensity index: t(11) = −11.808, p < 0.001; middle: t(11) = −7.132; p < 0.001; ring: t(11) = −11.874, p < 0.001; low intensity vs high intensity index: t(11) = −10.750, p < 0.001; middle: t(11) = −2.643; p = 0.023; ring: t(11) = −6.524, p < 0.001), and in purely thermal condition (no stimulation vs low intensity index: t(11) = −8.165, p < 0.001; middle: t(11) = −9.399; p < 0.001; ring: t(11) = −7.697, p < 0.001; low intensity vs high intensity index: t(11) = −2.101, p = 0.059; middle: t(11) = −3.229; p = 0.008; ring: t(11) = −5.386, p < 0.001). Thus, our stimulation intensities produced monotonic increases in the skin temperature of each finger, as expected (Table 1).

Next, we checked whether participants correctly *perceived* the different stimulations, by comparing magnitude ratings. Ratings increased with intensity for each finger both in the thermo-tactile (no stimulation vs low intensity index: t(11) = −6.191, p < 0.001; middle: t(11) = −8.456; p < 0.001; ring: t(11) = −3.761, p = 0.003; low intensity vs high intensity index: t(11) = −7.131, p < 0.001; middle: t(11) = −7.529; p < 0.001; ring: t(11) = −6.980, p < 0.001), and also in purely thermal condition (no stimulation vs low intensity index: t(11) = −7.288, p < 0.001; middle: t(11) = −5.998; p < 0.001; ring: t(11) = −6.425, p < 0.001; low intensity vs high intensity index: t(11) = −5.463, p < 0.001; middle: t(11) = −9.798; p < 0.001; ring: t(11) = −5.777, p < 0.001). Thus, varying intensity of stimulation induced concomitant variations in warmth perception, when each finger was stimulated individually (Table 1).

#### Uniformity judgement task

Our core scientific questions were 1) whether TR illusion was present in each of the thermo-tactile and purely thermal conditions and 2) whether the TR illusion differed in strength between these conditions.

First, a manipulation check assessed whether thermal shading was effective in influencing skin temperature of the middle finger. A 2 (Tactile condition: thermo-tactile, purely thermal) × 2 (Spatial pattern: uniform, non-uniform) repeated measures ANOVA on the difference between the middle finger and the average of the index and ring fingers skin temperature showed a significant main effect of Spatial pattern (F(1, 11) = 129.883; p < 0.001; η^2^ = 0.922), no significant effect of Tactile condition (F(1, 11) = 0.028; p = 0.871), and no interactions between the factors (F(1, 11) = 0.004; p = 0.948). The main effect arose because the difference between the middle finger and the other fingers was significantly higher in the non-uniform (mean ± SD: 2.0 °C ± 0.8 °C, tactile conditions averaged) than in the uniform stimulation condition (mean ± SD: 0.3 °C ± 0.8 °C, tactile conditions averaged), as predicted.

**Figure 3:**
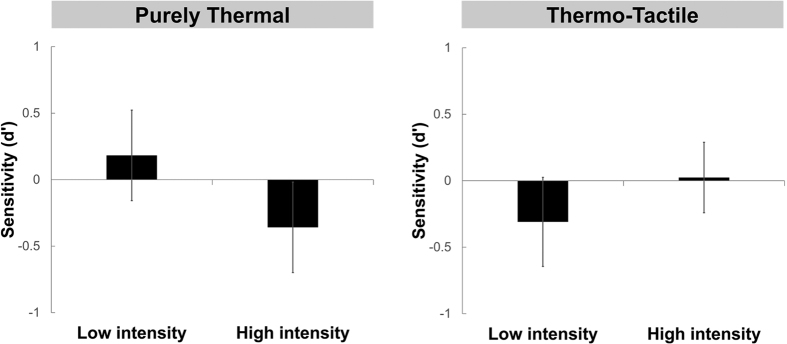
Thermal uniformity perception. Sensitivity (d′) measures in the purely thermal and thermo-tactile conditions. Performance was very poor in both experimental conditions, confirming a TR effect. No significant difference was found in sensitivity values between purely thermal and thermo-tactile conditions. Error bars show SE across participants.

We then tested whether the TR was present in both thermo-tactile and purely thermal conditions. A signal-detection approach was used, based on previous studies^5^. A hit was defined as a “uniform” response when the uniform thermal pattern was presented, while the false alarm was defined as a “uniform” response when the non-uniform thermal pattern was delivered by shading the middle finger. Sensory discriminability, d′ calculated as z(P_HIT_) − z(P_FA_), was then estimated from the hit rate and false alarm rate. Performance in detecting non-uniformity was very poor in both thermo-tactile and in the pure thermal condition (Fig. 3). Ten out of twelve participants in the thermo-tactile condition and nine out of twelve participants in the purely thermal condition showed a d′ lower than 1. Separate t-tests for each condition and intensity indicated that d’ scores were not significantly different from zero (thermo-tactile low intensity t(11) = −0.923, p = 0.376; thermo-tactile high intensity t(11) = 0.091, p = 0.930; purely thermal low intensity t(11) = 1.080, p = 0.303; purely thermal high intensity t(11) = −1.085, p = 0.301). Thus, participants were unable to detect thermal non-uniformity caused by middle-finger shading, confirming a TR illusion. A 2 (Tactile condition: thermo-tactile, purely thermal) × 2 (Intensity: low, high) repeated measures ANOVA was performed on the d’ values to compare the perceptual discriminability between thermo-tactile and purely thermal conditions (Fig. 3). The analysis revealed no main effect of Tactile condition (F(1, 11) = 0.029; p = 0.867), no main effect of Intensity (F(1, 11) = 0.44; p = 0.521) and no interaction between factors (F(1, 11) = 2.182; p = 0.168). We therefore found no evidence that TR experience was modulated by touch.

### Experiment 2

#### Thermal intensity perception

A manipulation check using a spot infrared thermometer confirmed that the thermal shading was effective at modulating skin temperature of the middle finger. A 2 (Tactile condition: thermo-tactile, purely thermal) × 2 (Spatial pattern: uniform, non-uniform) repeated measures ANOVA on the difference between the middle finger and the average of the index and ring fingers skin temperature confirmed a significant main effect of Spatial pattern (F(1, 11) = 63.075; p < 0.001; η^2^ = 0.852). No significant effect of Tactile condition (F(1, 11) = 3.539; p = 0.087), and no interactions between the factors (F(1, 11) = 0.209; p = 0.656) emerged. The main effect of Spatial pattern arose because during non-uniform stimulation the difference between the middle finger and the other fingers was significantly higher (mean ± SD: 2.3 °C ± 0.6 °C, tactile conditions averaged) than in the uniform stimulation condition (mean ± SD: 0.4 °C ± 0.7 °C, tactile conditions averaged). In essence, this data confirmed in each subject the same pattern of results found in our more detailed stimulus validation using thermal imaging.

**Figure 4:**
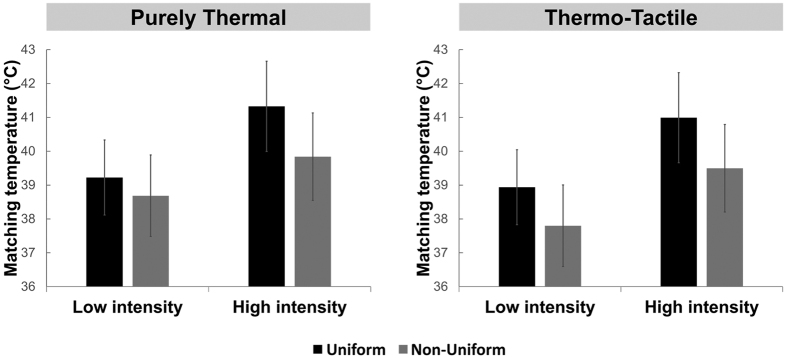
Thermal intensity perception. Participant reported when the thermode on the forehead reached the same temperature as the overall thermal sensation across index, middle and ring fingers. Overall intensity of physically non-uniform stimulations (middle finger shade present) was judged less intense than uniform patterns. No significant difference was found between purely thermal and thermo-tactile conditions. Error bars show SE across participants.

To analyze the overall intensity judgements, the perceived matching temperature in each condition was inserted in a 2 (Tactile condition: thermo-tactile, purely thermal) × 2 (Intensity: low, high) × 2 (Spatial pattern: uniform, non-uniform) repeated measures ANOVA (Fig. 4). This analysis showed no main effect of Tactile condition (F(1, 11) = 0.631; p = 0.444) but a significant main effect of both Intensity (F(1, 11) = 17.176; p = 0.002; η^2^ = 0.610) and Spatial pattern (F(1, 11) = 12.599; p = 0.005; η^2^ = 0.534). All interactions between factors were non-significant (p > 0.258). The main effect of intensity arose because, as expected, participants perceived high intensity of stimulation as significantly warmer (mean ± SD: 40.4 °C ± 4.6 °C, tactile and spatial pattern conditions averaged) than the low intensity of stimulation (mean ± SD: 38.7 °C ± 3.9 °C, tactile and spatial pattern conditions averaged). Crucially, the main effect of spatial pattern arose because a physically non-uniform pattern was perceived as significantly less intense (mean ± SD: 39 °C ± 4.3 °C, intensity and tactile condition averaged) than the physically uniform pattern (mean ± SD: 40.1 °C ± 4.3 °C, intensity and tactile condition averaged both in the thermo-tactile and the purely thermal condition). The perceived intensity was not significantly affected by touch.

### Experiment 3

#### Thermal spatial localization

First, we validated our method of stimulation, as in experiment 1, by computing the difference between post-stimulation and pre-stimulation skin temperature, and directly comparing the temperature gain for low vs high intensity, as for experiment 1. We confirmed clear differences in skin temperature between low vs high intensity (index: t(11) = −10.064, p < 0.001; middle: t(11) = −12.377; p < 0.001; ring: t(11) = −10.308, p < 0.001).

**Figure 5:**
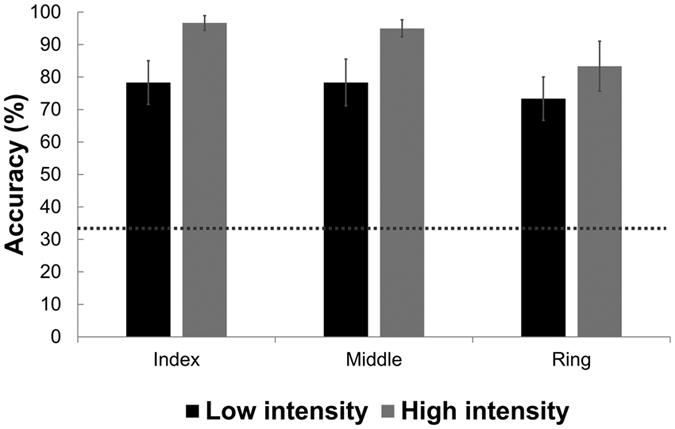
Thermal localization. Participant reported whether the thermal stimulation was delivered on the index, middle or ring finger. Overall accuracy is significantly different from chance level (indicated by a dashed line). Error bars show SE across participants.

Next, we analyzed accuracy of finger localization judgements for each finger stimulated, and for each intensity. Localization accuracy was always significantly better than the chance level of 33% (all p < 0.008 after Bonferroni correction for 6 tests). Accuracy rates, and a detailed breakdown of error types are shown in Fig. 5 and Table 2. Thus, we conclude that our radiant heat stimuli could be localized reliably to individual fingers. Detailed analysis of the *pattern* of localization errors showed that mislocalization to adjacent fingers was more frequent than to non-adjacent fingers (Table 2).

## Discussion

Here we describe for the first time a purely thermal TR, in the absence of any specific tactile stimulation: the sensation induced by purely thermal TR was indistinguishable from that induced by physically uniform stimulation, and also indistinguishable from the canonical tactile TR (experiment 1). The mechanisms underlying pure thermoceptive TR appear similar to those previously described for thermo-tactile TR^5^. Alternative explanations based on poor localization of thermal stimuli were rejected, since these radiant heat stimuli were localized rather accurately to individual fingers.

TR has been classically explained as a dominance of highly-weighted tactile information, over lower-weighted thermal signals in forming an integrated thermo-tactile percept. Evidence for this tactile-thermal integration comes from the fact that the illusory thermal sensation was reduced when the middle digit was lifted off the stimulator^4^. That result suggested an important multisensory component in classical TR. We have demonstrated that thermal signals are sufficient to produce TR effects, and that tactile contact is not necessary. Further, we found no evidence that tactile contact modulates the TR effect. Our results suggest that the basic mechanism underlying TR may arise from within the thermoceptive system, rather than from interactions between thermoception and mechanoception.

An alternative account of TR was given by Ho *et al*.^5^, based on serial processing of temperature and touch, rather than an integration of two parallel signals. Ho and colleagues proposed that, in a first stage, spatial summation tends to homogenize thermal percepts across multiple stimulated areas, producing an overall intensity percept proportional to the stimulated area. At a second stage, this intensity is then referred or attributed, on the basis of touch, to individual body parts – in this case the middle finger. Our results suggest that the first stage occurs within the thermoceptive system, and may be sufficient to explain our data.

Warm and cold thermoreceptors are fundamental in sensing external environmental temperatures in the innocuous range. The physiology of thermal processing is well known. When a purely thermal stimulus, as radiant warmth, is delivered to the skin, temperature-specific receptors in the skin are activated^17^. In the case of our stimulations, where skin temperature was increased of about 3 °C from baseline, unmyelinated, low threshold C fibers projecting to the Lamina I dorsal horn were presumably activated^18^. Then second order neurons transmit the information to the thalamus, which in turn projects to the cortex, primarily the insula^2^. Additional classes of warmth receptors have also been identified^18^,^19^. However, these, like classical nociceptors respond only at higher temperatures (39–51 °C)^18^,^19^,^20^, beyond the range studied here (30–35 °C).

When we touch an object, the sensations generated by thermal receptors are perceptually attributed to the object itself 21. Thus, although thermal perception is fundamentally interoceptive, the experiences it generates often have exteroceptive content. For example, we perceive the mug of tea as hot, though the receptors that drive this perception are, of course, located in the fingertips, not in the mug, and the thermal percept depends entirely on the fact that our fingers are in mechanical contact with the mug. The binding of sensory inputs to source objects is a ubiquitous feature of perception systems^22^,^23^. The possibility that touch guides thermal object perception was first suggested by the foundational work of Ernst Weber^3^. Weber observed that, in the absence of touch, the skin felt similarly warm when heated either by blood from within the body or by a radiant thermal source from outside the body. Thus the brain uses tactile contact between the skin and an external object to attribute the warm sensation to the external object rather than to the body itself. Essentially the same argument is used in the classical account of TR^5^. Attributing the thermal and tactile sensations on the three fingers to a common, spatially-extended source object triggers a powerful process of perceptual integration^24^. In this integration process, the tactile sensations receive a relatively higher weighting than the thermal sensations. Tactile uniformity over-rides thermal non-uniformity, producing the TR illusion of a homogenous temperature. Green^4^ reported that lifting the neutral middle finger to break tactile contact abolishes TR. That is, a change in purely tactile input produced an illusory change in thermal perception. This result suggests that the homogeneity of tactile stimulation across the three fingers may explain the high weighting given to touch.

Importantly, these previous accounts assume that conscious perception occurs only subsequent to these processes of multisensory integration and object attribution. Conscious access to purely thermal sensation is precluded, because thermo-tactile percepts are assumed to be metameric: when participants are asked to judge thermal uniformity, they in fact report a multisensory thermo-tactile percept of the external object. Our results do not deny that source object attribution and multisensory integration play important roles in TR, but they do suggest that these mechanisms are not necessary. TR can equally occur in the absence of tactile inputs signaling an external object.

Since TR is possible without source object attribution, we can ask what features of the organisation of the thermoceptive pathway itself could underlie the effect. We consider four possibilities in turn: processing bandwidth, spatial resolution, thermal “filling-in”^25^, and spatial summation^7^,^8^.

First, our purely thermal TR could simply reflect limited attentional capacity^26^. People cannot perceive more than two touches in parallel^27^. Thermoception may be similarly limited. However, such bandwidth accounts cannot readily explain our results. First, our stimuli were delivered over an extended period of time, allowing participants enough time for allocating selective attention to each finger in turn. Second, a defining feature of attentional systems is that intense or salient stimuli nevertheless “break through” the limits of attention. When several stimuli are presented in parallel, a stimulus of lower or higher intensity than the others will *pop out* and automatically attract selective attention^28^. If perceptual/attentional capacity explained our results, then non-uniformity detection should improve at higher thermal stimulation intensities, because the unstimulated middle finger should more readily pop out. In fact, we found a non-significant trend in the opposite direction, casting doubt on attentional explanations of our effect (Fig. 3).

Second, purely thermal TR could reflect the thermoceptive system’s *low spatial resolution*^15^,^16^,^29^. Poor thermal spatial resolution would imply a single overall percept when three fingers are stimulated, losing information about local variation that underlies detection of non-uniformity. Classical studies support this view: indeed, people reported feeling warmth on the stomach when radiant heat was applied to the lower back^15^. Pritchard^30^ commented that “*it is only when the … stimulus … involves deformation of the skin that accurate localisation is possible*”. The spatial resolution for non-contact radiant warmth was estimated between 4.5 cm and 15 cm on the forearm and around 14 cm on the back^15^,^16^. Our results show that localization of radiant heat to a single finger was surprisingly accurate. One might argue that localization can be inferred by the difference between the thermal intensities perceived on each finger. Indeed, people can accurately perceive discrepancies in thermal sensations across different fingers^6^. However, participants could only use differences in perceptual intensity to localise a thermal stimulus if they can (1) perceive that the fingers are not uniformly warm and (2) correctly identify which fingers feel warmer, and which feel less warm. Therefore, if people adopt intensity discrepancies to perform thermal localisation, they should, in principle, also be able to detect the uniformity of a pattern of thermal stimulation across the fingers. However, our results do not support this line of reasoning. We showed that participants could not perceive any non-uniform pattern when presented with a warm-neutral-warm pattern of stimulation, even though they could readily localize the same degree of warmth when delivered to a single finger. Thus, poor spatial resolution of warm sensations cannot readily explain our results. Specific perceptual mechanisms related to thermal *patterns* across multiple fingers appear necessary.

Another possible explanation of TR is based on a process known as “filling-in”. The warm input to the outer fingers would lead to filling-in a similar warm sensation at the middle finger, despite absence of thermal stimulation. In vision, percepts such as the Troxler effect^31^ are based on perceived homogeneity due to loss of local stimulus detail. Low-level and high-level theories have been proposed. According to low-level theories, early visual cortex neurons tuned to different dimensions, such as orientation and colour, may interact to produce neural activity in the absence of physical stimulation^32^. According to high-level theories, a cognitive mechanism that assumes homogenous objects leads to a conceptual or symbolic extrapolation of detail from areas of stronger to weaker perceptual signal^33^. The latter account strongly recalls the attribution of multisensory inputs to a homogenous thermo-tactile source object in TR. Further, a thermal completion mechanism would predict that the physical intensity applied to the stimulated fingers is “copy-pasted” from the stimulated index and ring fingers to the non-stimulated middle finger, resulting in an unchanged, or at least not decreasing, percept of overall intensity^5^. Our results do not support this “filling-in” hypothesis: the perceived overall intensity was significantly *reduced* in the non-uniform condition compared to the uniform condition.

Finally, *spatial summation* occurring within the thermoceptive system might readily explain our results. Classically, spatial summation is demonstrated by a decrease in the thermal detection threshold, or increase in suprathreshold intensity perception, when stimulating larger, rather than smaller skin regions. Spatial summation within the warm afferent channel is strong^7^,^8^. During TR, spatial summation would imply a stronger sensation of warmth in the physically uniform stimulation, in which three fingers are stimulated, than in the non-uniform stimulation, where only two are stimulated. Indeed, participants in Ho *et al*.’s thermo-tactile experiment^5^ perceived a lower overall intensity when the middle finger received no thermal stimulation (the TR condition), than when it was stimulated, consistent with the predictions of spatial summation. Our study confirmed this hypothesis. Participants perceived a lower overall intensity for non-uniform patterns compared to uniform patterns, even when stimulation was purely thermal. Classically, somatosensory neurons integrate all the inputs in their receptive field. Neurons with spatially–extended, multi-digit receptive fields could thus underlie spatial summation^34^,^35^. Our result suggests that thermal referral effects are not dependent on tactile localization, and may arise within the thermoceptive system. One may speculate that the thermoceptive system contains neurons with finger-specific receptive fields, which may then converge on higher-level neurons that summate their inputs, and thus have multi-finger receptive fields. Our result leads to the intriguing idea that localization of a thermal stimulus occurs at the first level, where digit-specific information is available. In contrast, information about the overall pattern of thermal intensities, as in our uniformity judgements for example, occurs only at the second level, where digit-specific information is not available.

Most previous studies of TR involved thermo-tactile stimuli. When tactile stimuli are applied on the fingers of one hand, the tactile signals are initially processed separately. Next, the variability among the different fingers is computed. If variability is low, then a homogenous tactile object is assumed, and the tactile signals from the three fingers are combined. The thermal processing pathway lacks such a sophisticated object detection system. Rather, a degree of homogenization might operate automatically, and at an early processing stage, to produce a global representation, with little local detail. When both thermal and tactile signals are available, uniformity of stimulation across fingers is based on an integrated percept reflecting a unified average of both, rather than on a unisensory source. The relative weightings of tactile and thermal information in multisensory integration may explain the apparent discrepancy between Green’s result^4^, and ours. In his experiment, raising the middle finger from the stimulator produced tactile signals of non-uniformity, which lead to a thermal percept of non-uniformity. In our shadow condition, the thermal conditions were identical to Green’s middle-finger raised condition, but the tactile conditions were quite different. In particular, the non-homogenous tactile signals of Green’s study were absent in our study. That is, homogeneity of tactile input appears essential for the illusion, although positive presence of a tactile object is not essential. TR requires either all stimulated fingers in contact or all stimulated fingers contact-free. We speculate that the thermal experience of traditional TR is exteroceptive and is attributed to an external object. Conversely, in our purely thermal TR, the thermal experience may be more interoceptive, and might be attributed to one’s own body. This speculation could be directly tested in the future, by repeating our experiment using much lower levels of radiant heat, below the threshold for detecting an external heat source.

In conclusion, low-level mechanisms of spatial summation within the thermoceptive system seem sufficient to explain an illusion that had previously been interpreted as reflecting multisensory, cognitive processes of object perception.

## Additional Information

**How to cite this article**: Cataldo, A. *et al*. Thermal referral: evidence for a thermoceptive uniformity illusion without touch. *Sci. Rep.* **6**, 35286; doi: 10.1038/srep35286 (2016).

## Figures and Tables

**Table 1:** Individual finger temperature perception data.

		Index Finger			Middle Finger			Ring Finger		
		No	Low	High	No	Low	High	No	Low	High
*Skin Temperature* (*°C*)										
Thermo-tactile	*M*	−0.07	1.55	3.20	0.39	1.65	2.07	0.10	1.80	3.01
	*SD*	0.52	0.52	0.61	0.70	0.62	0.71	0.67	0.57	0.48
Purely Thermal	*M*	0.04	1.58	2.03	−0.28	1.10	1.75	−0.14	1.30	2.27
	*SD*	0.61	0.57	0.65	0.66	0.59	0.68	0.80	0.63	0.37
*Magnitude Estimates* (*from 0 to 10*)										
Thermo-tactile	*M*	0.33	2.50	5.29	0.29	2.46	4.75	0.38	1.88	5.04
	*SD*	0.39	1.17	1.48	0.45	0.78	1.27	0.43	1.26	1.71
Purely Thermal	*M*	0.50	3.21	5.67	0.50	3.25	5.25	0.25	1.67	3.25
	*SD*	0.85	1.20	1.74	0.43	1.66	1.75	0.34	0.72	1.16

Differences between post-stimulation and pre-stimulation skin temperature (degrees) and magnitude estimates (scale unit) in function of the radiant stimuli intensity. No = no stimulation; Low = low intensity; High = high intensity.

**Table 2:** Confusion matrix of the accuracy in the localization task (Experiment 3).

		*Reported finger*		
		Index	Middle	Ring
*Stimulated finger*	Index	87.5 (1.3)	9.2 (1.0)	3.3 (0.5)
	Middle	2.5 (0.5)	86.7 (1.2)	10.8 (1.2)
	Ring	2.5 (0.6)	19.2 (1.8)	78.3 (2.1)

For each finger stimulated, the percentage (and standard deviation across participants) of each response is given. Values on the diagonal are correct responses.
